# Applied RNAi: from fundamental research to therapeutic applications

**DOI:** 10.3389/fgene.2014.00398

**Published:** 2014-11-18

**Authors:** Bernard Mari, Barbara Bardoni

**Affiliations:** Institut de Pharmacologie Moléculaire et Cellulaire, Centre National de la Recherche Scientifique, UMR 7275 CNRS/UNSASophia Antipolis, France

**Keywords:** RNAi, miRNA, RNAi-based therapy, pluripotency, infection, Cancer

One of the most exciting aspects of the genomic era during the last fifteen years has been the discovery of RNA interference pathways and their key function in gene regulation in eucaryotes. RNAi has rapidly invaded our laboratories and has become the gold standard technique to investigate the function of a given gene. In parallel, intensive research on microRNAs (miRNAs) has underlined their wide roles in development, homeostasis and diseases processes. However, the road toward the therapeutic use of RNAi in human has proven to be more tortuous than anticipated with difficulties in minimizing several off-target effects, and it is becoming clear that this strategy can only succeed in a limited number of diseases at present.

As underlined in the preface, the editors, Patrick Arbuthnot and Marc S. Weinberg do not aim to propose a comprehensive book describing the large background in the field and general protocols for gene silencing, as already performed by several authors but have rather chosen to highlight some of the most exciting aspects of current RNAi research. Indeed, the book is organized in twelve chapters focused on different subjects ranging from fundamental biological aspects to specific topics on RNAi-based therapy.

Chapter I, “Overview of biogenesis and applications of miRNA” by Arbuthnot and Weinberg is an excellent introduction to the field. The authors review precisely and concisely all the aspects of miRNA biogenesis and the discoveries concerning miRNA function and role in cell and organs as well as their wide potential therapeutic applications.

The second Chapter, “Non-canonical miRNA biogenesis and function” describes the different forms of non-canonical biogenesis of miRNAs as well as new emergent families of small RNAs. This chapter also addresses non-canonical miRNA functions such as activation of translation or transcriptional gene regulation and discusses recent investigations regarding interactions between miRNAs and long non-coding RNAs and the potential role of miRNAs in the extracellular environment. A third chapter “Non-coding RNAs and the Epigenetic Control of Gene Expression” logically pursues this description about the emerging concept of a RNA-based epigenetic regulatory network whilst remaining quite superficial on these issues. This more fundamental section appears complete and overall well organized, with minimal overlap on general principles governing miRNA biogenesis and functions.

Having presented this detailed background on the subject, the following chapters deal with RNAi and miRNA technologies. Chapter 4, “From mice to men: toward the clinical translation of miRNA technologies for somatic cell reprogramming” is a very interesting section, not only describing the role of miRNA in iPS generation but also the approaches that have been developed by different groups to identify miRNAs involved in pluripotency and their potential use as new tools in regenerative medicine.

Three chapters focus on RNAi, miRNAs and virus infection, covering very different, yet complementary fields with focus on “Systems biology tools to understand the role of host miRNAs in infection” (chapter 5), a review on “Harnessing RNAi for the Treatment of Viral Infections” (chapter 9) and a specific chapter on “Roles of miRNAs in Cancers Associated with Human Tumor Viruses (chapter 10). Because RNAi represents an ancestral cellular defense mechanism against various viruses, whilst on the other hand many viruses express their own miRNAs, this section provides a very interesting and quite complete picture of the complex defense and counterattack molecular mechanisms developed by cells and viruses. The authors also underline the efforts to improve delivery and decrease unwanted side effects of RNAi as therapeutic tools against virus, which may soon provide efficient weapons against devastating diseases such as the current Ebola virus outbreak.

A specific chapter (chapter 11) is devoted to “MiRNAs as cancer biomarkers” giving a good overview of the considerable interest in this field. It would however have been important here to strongly warn against the heterogeneity of the studies reporting circulating miRNA cancer biomarkers because of several potential technical biases.

The following chapter (chapter 12) describes the role of miRNAs in Trinucleotide Repeat Expansion Disorders. The common basis of these diseases is the presence of an expanded sequence in the protein or in the mRNA, but the function of the genes that are involved is very different as well the brain regions that are involved in the disorder. For this reason, the main interest of the role of miRNAs in these kinds of disorders is the possibility to use them for down-regulation of the abnormal transcripts that are the cause of neurodegeneration.

Given the number of studies on the role of miRNAs in cancer and nervous system, it is however frustrating not to find additional information on these key topics, for example, the relevance of those molecules in the formation and plasticity of the synapse with potential implication in neurological and psychiatric disorders.

Finally, a key section of the book corresponds to three chapters dealing with “Synthetic miRNA Blocking Agents” (chapter 6), “Exploiting miRNAs to Regulate Transgene Expression” (chapter 7) and “Use of Artificial miRNAs for Gene Silencing” (chapter 8) that very clearly describe and discuss the various experimental and therapeutic applications of miRNAs. The chapter related to miRNA blocking agents offers a very complete and precise view of this complex area and should be read by any investigator initiating *in vitro* or *in vivo* studies with miRNA inhibitors.

In conclusion, this book is well written, each chapter is not too long and in general the reading is easy with a few discrepancies between the quality of the different sections as mentioned above. Overall we believe that this book can be very useful for students as well for researchers starting to work in this subject. Some basic studies on this subject are well developed, the main applications and limitations of RNAi and miRNA-base technology are presented and a large amount of literature is summarized in tables, simplifying the search of studies of interest.

## Conflict of interest statement

The authors declare that the research was conducted in the absence of any commercial or financial relationships that could be construed as a potential conflict of interest.

